# Modeling Individual Subtests of the WAIS IV with Multiple Latent Factors

**DOI:** 10.1371/journal.pone.0074980

**Published:** 2013-09-13

**Authors:** Dennis J. McFarland

**Affiliations:** Laboratory of Neural Injury and Repair, Wadsworth Center, New York State Department of Health, Albany, New York, United States of America; Cuban Neuroscience Center, Cuba

## Abstract

Performance on a cognitive test can be viewed either as measuring a unitary function or as reflecting the operation of multiple factors. Individual subtests in batteries designed to measure human abilities are commonly modeled as a single latent factor. Several latent factors are then used to model groups of subtests. However these latent factors are not independent as they are related through hierarchical or oblique structures. As a result, the simple structure of subtest performance results in complex latent factors. The present study used structural equation modeling to evaluate several multidimensional models of the Wechsler Adult Intelligence Scales- fourth edition (WAIS-IV) subtests. Multidimensional models of subtest performance provided better model fit as compared to several previously proposed one dimensional models. These multidimensional models also generalized well to new samples of populations differing in age from that used to estimate the model parameters. Overall these results show that models that describe subtests as multidimensional functions of uncorrelated factors provided a better fit to the WAIS-IV correlations than models that describe subtests as one dimensional functions of correlated factors. There appears to be a trade-off in modeling subtests as one dimensional and modeling with homogeneous latent traits. More consideration should be given to models that include multiple uncorrelated latent factors as determinants of the performance on a given subtest. These results support the view that performance on any given cognitive test is potentially the result of multiple factors. Simple structure may be too simple.

## Introduction

One view of the determinants of performance on standardized cognitive tests holds that performance is potentially the result of multiple factors [1], [2]. For example, Milberg and associates [3] describe a number of distinct processes that might limit performance on single subtests of the Wechsler Adult Intelligence Scales- revised (WAIS-R). This view is supported by examination of the impact of different patterns of brain pathology on test performance. In contrast, some researchers hold that a single factor (g) accounts for most of the variance in cognitive test performance [4]. This view holds that clinical interpretation should be primarily at the level of general intelligence and is supported by factor analytic studies of test performance.

There is a problem with the factor analysis of abilities tests as proposed by Hart and Spearman [5] due to the fact that these models have more latent variables than observed variables [6], [7]. Thurstone [8] advocated the concept of simple structure as a goal in factor analysis. By simple structure Thurstone [8] was referring to solutions with a large number of zeros or nearly vanishing entries in the factor matrix (ie., each test should have nonzero loadings on as few factors as possible). Thurstone [8] considered simple structure to be a psychologically meaningful solution to the factor indeterminacy problem. Other authors have also viewed simple structure to be essential [9]. While Thurstone [8] considered it reasonable to assume that any given mental test might be determined by a single psychological factor not all theorists agreed [10].

Current practice in modeling test batteries such as the Wechsler Adult Intelligence Scales- fourth edition (WAIS-IV) is based on this concept of simple structure. For example, the solution provided in the WAIS-IV manual (Figure 5.3, [11]) has only one subtest loading on more than one factor. Likewise, alternative models applied to the WAIS-IV standardization sample suggested by other authors have but one WAIS-IV subtests loading on more than one factor [12], [13]. These simple solutions were obtained by using either hierarchical or oblique factor models. An exception is the work of Gignac [14] who modeled the correlations from the WAIS-III standardization sample using a “nested” factor model with a “g” factor orthogonal to group level factors and loading directly on each subtest. Gignac [14] found that modeling g as a first-order factor resulted in better model fit and less ambiguous factor loadings.

Smith and associates [15] have argued for the use of homogeneous constructs. They contend that oblique and hierarchical models produce complex latent variables that do not represent homogeneous constructs. In addition, as pointed out by Gignac [14], higher-order models imply that the effects of the higher level factor (i.e., g) are not direct, but rather are mediated by group level factors. Finally, as noted by Gignac [14], oblique models imply that the covariance between factors is multidimensional. That is, if several factors are correlated, then their variance can be partitioned into orthogonal common and unique components (i.e., the common component of the pair and the two unique components). In the case of a hierarchical model this common component is the same for all factor pairs. In the case of oblique models this common component differs for each pair. Thus, the covariance of an oblique model with n factors can be partitioned into an orthogonal model with n!/(2*(n–2)!) factors (eg., the model of Ward and associates [13], with five oblique factors, is equivalent to a model with 15 orthogonal factors).

Although hierarchical models imply that subtest performance is one dimensional, with higher order effects mediated by lower order processes, models of cognitive architecture often postulate multiple processes that determining observed behavior. For example, Franzen [16] suggests that Neuropsychological test performance is determined by factors related to the stimulus, processing and the response. Shiffrin and Schneider [17] state that “automatic and controlled processes are used in combination in all tasks”. Barrett and Kurzban [18] have argued that mental processes “consist of multiple specialized systems, rather than a single general purpose one.” Anderson and associates [19], have postulated four cognitive processes operating during solution of simple arithmetic problems to account for activations occurring in multiple brain areas. Studies of event-related potentials have been interpreted as reflecting the operation of multiple cognitive subsystems [20]. As noted earlier, Milberg and associates [3] describe a number of distinct processes that might limit performance on single subtests of the WAIS-R. As these examples illustrate, it is common practice to hypothesize multiple determinants of performance of single tasks. While it is possible that a single factor produces individual differences in these multiple cognitive processes, it is also possible that each cognitive process is subject to independent variation across individuals.

The present study evaluated the hypothesis that performance on any mental test necessarily involves multiple cognitive processes [21]. If this is so, then it would be expected that structural models that allow for multiple factor loadings on each of the WAIS-IV subtests should provide a better fit than those with simple structure. In order to test this hypothesis models with loadings of all subtests on all factors were evaluated. These multidimensional models were compared to several models previously proposed to account for the correlations between the WAIS-IV subtests. Because these multidimensional models have a large number of free parameters particular emphasis was placed on generalizing the parameter estimates to new data.

## Methods

### Participants

This study used the data reported for the standardization sample of the WAIS-IV [11]. Three samples were constructed consisting of data for individuals between 20 and 54 years of age (Tables A.3 through A.7, n = 1000), individuals between 16 and 19 years of age (Tables A.1 and A.2, n = 400), and individuals between 55 and 69 years of age (Tables A.8 and A.9, n = 400). The publisher states that the sample was stratified to match the United States population based on the demographic variables of age, sex, education level, race/ethnicity, and geographic region.

### Analyses

Three models of WAIS-IV structure obtained from the literature were compared with several models that had multiple orthogonal factor loadings for each subtest. The 20–54 year-old sample from the WAIS-IV standardization data was used for parameter estimation and two additional samples were used for cross-validation with parameter values fixed to the estimates obtained from the 20–54 year-old sample.

Correlations matrices included all 15 subtests of the WAIS-IV. For each sample, tabled values were combined by first applying Fisher’s z transform, then averaging all of these values for each pair of subtests in a sample, and then taking the inverse transform to produce average r values. All correlations were positive and all tabled values were based on the same number of participants (i.e., 200) so that these factors were not considered in averaging r values.

All analyses were done with the SAS CALIS procedure [22] using default settings. All latent factors were set equal to 1 (except for those defined by a hierarchical structure) as recommended by Anderson and Gerbing [23]. Comparison models included the model presented in Figure 5.2 of the WAIS manual [11] (subsequently referred to in Tables as Wechsler 2008), the model presented in Figure 3 of Benson and associates [12] (Benson 2010) and the model presented in Figure 4 of Ward and associates [13] (Ward 2011). These are the models with the best fit statistics reported by each author for the 15 subtests of the WAIS-IV. The models of Wechsler [11] and Benson and associates [12] are hierarchical models while that of Ward and associates [13] is an oblique model. The Wechsler [11] model has four first-order factors (ie., verbal comprehension, perceptual reasoning, working memory and processing speed) and a second-order *g* factor. Both Benson et al. (2010) and Ward et al. (2011) describe their models as versions of the Cattell-Horn-Carroll (CHC) model with five first-order factors (ie., crystallized ability, visual processing, fluid reasoning, short-term or working memory and processing speed). The Benson and associates [12] model has a second order *g* factor while the Ward and associates [13] model estimates a correlation between each pair of these factors. Additional models were evaluated with 1, 2, 3, 4, 5 and 6 orthogonal factors (4,5, and 6 independent) that had loadings for every factor on each scale. Three final models were evaluated. These were a modification of the Benson and associates [12] model that represented the general factor as a “nested” factor [14] uncorrelated with the other factors (ie., all factors are first-order)(Nested CHC), a model starting with this nested model with added modifications suggested by the CALIS diagnostic indices (Modified CHC), and a model starting with the 6 orthogonal factors that was modified according to these diagnostic indices (Modified 6 indep).

Modifications of the two models as suggested by the CALIS diagnostic indexes (i.e., Modified CHC and Modified 6 indep) were done in steps with a single weight changed and the results subsequently evaluated prior to the next modification. Each step consisted of adding the single factor loading suggested by the Lagrange multiplier ranked largest, provided that this was projected to result in a significant change in the chi-squared (X^2^) statistic (p<0.05). If no addition was indicated by the Lagrange multiplier then the smallest change in the X^2^ statistic projected by the Wald test was eliminated, provided that this did not result in a significant change in the X^2^ statistic. This process continued if the actual changes in the X^2^ statistic and the diagnostic criteria met these same criteria.

Fit indices included chi-squared (X^2^), the goodness of fit index (GFI), the adjusted goodness-of fit index (AGFI), the standardized root-mean-square error (RMSEA), the Akaike information criterion (AIC) and Bentler’s comparative fit index (CFI). These indices were selected so as to provide a comparison with prior studies. These indices differ in how they deal with the trade-off between model accuracy and complexity. The fact that there has been a proliferation of such indexes attests to the difficulty of equating accuracy and complexity. This problem of model evaluation is also dealt with by the use of cross-validation [24]. Cross validation in the present study used the loadings for each factor on each subtest as estimated from the 20–54 year old correlations. These were applied separately to the correlation matrices from the 16–19 year old and 55–69 year old participants. Only the scale-specific effects (error) were estimated in the evaluation of models with cross-validation, as is also the case with the NULL model, which was also included.

## Results

A summary of fit indices for the various models applied to the 20–54 year old sample is shown in Table 1. The model presented in the WAIS manual [11] provides a reasonable fit but both that of Benson and associates [12] and Ward and associates [13] show improvements on all indices. The residual variance associated with the fluid factor of the Benson and associates [12] model initially produced a negative value so it was constrained to be greater than zero. These three models will be referred to as the comparison models. All indices improved as more factors were included in the multiple orthogonal factor models. The four orthogonal factors model fit this correlation matrix better than the three comparison models for the GFI, Akaike and CFI indices. The five orthogonal factor model fit better than the comparison models on all indices except the AGFI. With the addition of the sixth factor the parallel model outperformed the comparison models on all indices.

**Table 1:** Model Fit for the WAIS Standardization Data for Ages 20–54.

Model	df	X^2^	GFI	AGFI	CFI	RMSEA	Akaike’s
Null model	105	8697.69	0.2530	0.1463	0.0000	0.2862	8487.69
Wechsler 2008	80	398.39	0.9469	0.9203	0.9629	0.0631	238.39
Benson 2010	80	331.65	0.9567	0.9350	0.9707	0.0561	171.65
Ward 2011	78	263.11	0.9664	0.9483	0.9785	0.0487	107.11
4 independent	45	167.26	0.9775	0.9401	0.9858	0.0521	77.26
5 independent	30	95.48	0.9871	0.9483	0.9924	0.0467	35.48
6 independent	15	41.55	0.9945	0.9559	0.9969	0.0421	11.55
Nested CHC	74	298.59	0.9610	0.9368	0.9739	0.0551	150.59
Modified CHC	62	91.44	0.9881	0.9770	0.9966	0.0218	−32.56
Modified 6indep	47	52.59	0.9931	0.9824	0.9993	0.0109	−41.41

The modified CHC and Modified 6 independent models were the result of using the CALIS modification indices as described in the methods.

The nested model that modified the general factor in the Benson and associates [12] model to be an orthogonal factor (nested CHC model) had improved fit compared to the original model for all indices. However the Ward and associates [13] model outperformed the nested CHC model on all indices. The modified models (i.e., models produced with the CALIS modification indices) provided a better fit than the comparison models on all indices and outperformed all other models except for the six orthogonal factor model on the X^2^ and GFI indices, which do not adjust for model complexity. The modified six orthogonal factor model was the only model with a X^2^ that did not differ significantly from chance (p<0.2665).

A summary of the generalization of the factor loadings fitted with the 20–54 year old sample to the data from 16 to 19 year old participants is shown in Table 2. Because all models had the same number of degrees of freedom (i.e., only the subtest specific “error” was estimated), all fit indices showed the same rank ordering. The Benson and associates [12] model provides the best fit of the comparison models. The nested CHC model was slightly better. Both the five and six factor parallel models generalized to the younger participants better than the comparison models. The modified six orthogonal factor model generalized better than the comparison models and the five orthogonal factor model but was not as good as the six orthogonal factor model. All models had X^2^ values that differed significantly from chance.

**Table 2:** WAIS Standardization Data Generalized to Ages 16–19.

Model	df	X^2^	GFI	AGFI	CFI	RMSEA	Akaike’s
NULL	105	3018.82	0.2843	0.1821	0.0000	0.2637	2808.82
Wechsler 2008	105	239.36	0.9261	0.9156	0.9539	0.0566	29.36
Benson (2010)	105	205.17	0.9382	0.9293	0.9656	0.0489	−4.83
Ward (2011)	105	206.30	0.9366	0.9276	0.9652	0.0492	−3.70
4 independent	105	201.56	0.9353	0.9261	0.9669	0.0480	−8.44
5 independent	105	173.84	0.9451	0.9372	0.9764	0.0405	−36.16
6 independent	105	151.54	0.9531	0.9464	0.9840	0.0333	−58.46
Nested CHC	105	202.29	0.9380	0.9291	0.9666	0.0482	−7.71
Optimized CHC	105	187.62	0.9416	0.9333	0.9716	0.0444	−22.38
Optimized 6 uncorr	105	160.66	0.9501	0.9430	0.9809	0.0364	−49.45

A summary of the generalization of the factor loadings fitted with the 20–54 year old sample to the data from 55 to 69 year old participants is shown in Table 3. The Ward and associates [13] model provides the best fit of the comparison models. The nested CHC model was slightly better. Both the five and six parallel factor models generalized to the older participants better than the comparison models. The modified six orthogonal factor model generalized better than all other models. All models had X^2^ values that differed significantly from chance.

**Table 3:** WAIS Standardization Data Generalized to Ages 55–69.

Model	df	X^2^	GFI	AGFI	CFI	RMSEA	Akaike’s
NULL	105	3726.00	0.2255	0.1148	0.0000	0.2940	3516.00
Wechsler 2008	105	273.93	0.9160	0.9039	0.9533	0.0635	63.93
Benson 2010	105	253.71	0.9243	0.9135	0.9589	0.0596	43.71
Ward 2011	105	212.58	0.9341	0.9247	0.9703	0.0507	2.58
4 independent	105	217.03	0.9337	0.9242	0.9691	0.0517	7.03
5 independent	105	205.39	0.9369	0.9279	0.9723	0.0490	−4.61
6 independent	105	202.79	0.9387	0.9299	0.9730	0.0483	−7.21
Nested CHC	105	242.23	0.9276	0.9172	0.9621	0.0572	32.23
Modified CHC	105	222.19	0.9336	0.9241	0.9676	0.0529	12.19
Modified 6indep	105	199.54	0.9395	0.9308	0.9739	0.0475	−10.46

Overall these results show that modeling the WAIS-IV subtests as determined by multiple orthogonal factors provide a better fit of the data. The modified six orthogonal factor model is simpler and provided somewhat better performance on several of the fit indices from the 20–54 year old sample than the six orthogonal factor model. In addition, the modified six orthogonal factor model was the only model not significantly different from chance. However the original six orthogonal factors model generalized better to the cross-validation samples. Details of both modified models are discussed below.

The factor loadings estimated from the 20–54 year old sample associated with each subtest for the modified CHC model are shown in Table 4. In general, the structure of this model resembles that of the Benson and associates [12] model from which it was derived. Factor 1 has large positive loadings for every subtest and can readily be interpreted as a general factor (g) corresponding to the higher-order factor of Benson and associates [12]. Factor 2 has largest loadings on Vocabulary, Comprehension, Information and Similarities, as does the Benson and associates [12] Crystallized Abilities factor. It differs from the Benson and associates [12] model in that it includes a small loading on Picture Completion. Picture Completion involves knowledge of the form of visual objects. Like the verbal items loading on this factor, it can be described as relating to semantic knowledge (memory). Factor 3 has the largest loadings for Visual Puzzles, Block Design and Picture Completion, as does the Benson and associates [12] Visual Processing factor. In addition, Factor 3 also has loadings on Cancellation, Figure Weights, Matrix Reasoning and Symbol Search, all tasks that involve visual processing. Factor 4 has positive weights on Information, Vocabulary and Arithmetic. It does not correspond with the Fluid Reasoning factor of Benson and associates [12] from which it evolved. Factor five has loadings on digit span, letter-number sequencing and arithmetic, as does the short-term memory factor of Benson and associates [12]. In addition, factor 5 has loadings on Picture Completion, Cancellation and Vocabulary. Factor 6 has the largest loadings on Symbol Search, Coding and Cancellation and corresponds roughly to Processing Speed in the Benson and associates [12] model. Unlike Benson and associates [12] though, this model suggests that Processing Speed is a factor on other subtests, such as Picture Completion, Digit Span and Feature Weights.

**Table 4:** Weights for the Modified CHC Model.

Subtest	Factor 1	Factor 2	Factor 3	Factor 4	Factor 5	Factor 6
Block Design	0.6474		−0.4970			
Similarities	0.7044	0.4689				
Digit Span	0.6859			−0.1009	−0.6089	0.1022
MatrixReasoning	0.7158		−0.1336			
Vocabulary	0.6796	0.5690		0.2389	−0.1080	0.0616
Arithmetic	0.7704			0.1914	−0.1709	
Symbol Search	0.4853		−0.1419			0.6610
Visual Puzzles	0.6571		−0.4815			−0.0625
Information	0.6487	0.3909		0.3076		
Coding	0.5367					0.5746
L-N Sequencing	0.6656				−0.4047	
Figure Weights	0.7780		−0.1254			−0.1169
Comprehension	0.6985	0.4990				
Cancellation	0.3472	−0.1109	−0.1997			0.3981
PictureCompletion	0.4563		−0.3914			0.1589

The estimated factor loadings associated with each subtest for the modified six orthogonal factor model derived from the 20–54 year old sample are shown in Table 5. Factor 1 has large positive loadings for every subtest and can readily be interpreted as the general factor (g). Factor 2 has loadings on 12 of the fifteen subtests, so it also appears to be a general factor. Unlike Factor 1 however, it is a mixture of both positive and negative loadings. The positive loadings seem to be related to Processing Speed [12], with largest positive loadings on Symbol Search, Cancellation and Coding. The negative loadings seem to be related to Crystallized Ability, loading on Comprehension, Vocabulary, Similarities and Information. Implications of this pattern will be considered in the discussion. Factor 3 is also a contrast between four positive and 5 negative loadings. The negative loadings on visual puzzles, block design, and picture completion correspond to the perceptual reasoning factor of Wechsler [11]. The positive loadings on Coding, Symbol Search, Vocabulary and Digit Span do not clearly correspond to any of the factors in the comparison models. However their verbal content is in contrast to the visual-spatial nature of the subtests with negative loadings on this factor. Factor 4 is a contrast between positive loadings on Comprehension, Similarities, Picture Completion and Vocabulary with negative loadings on Arithmetic and Figure Weights. Factor 5 has positive loadings on Information, Vocabulary, Similarities and Comprehension, corresponding to crystallized ability [12]. It also has a positive loading on Picture Completion. As Picture Completion requires semantic knowledge (i.e., general knowledge about the world) of visual form it arguably requires crystallized ability. The negative loadings on Coding, Matrix Reasoning and Digit Span are all tasks relatively low in their requirements for semantic knowledge. Finally, factor six has negative loadings on Digit Span, Letter Number Sequencing, Arithmetic and Figure Weights, corresponding to the Working Memory factor of Wechsler [11]. All of these loadings except for Figure Weights also correspond to the Short-Term Memory factor of Benson and associates [12].

**Table 5:** Weights for the Modified 6 Independent Factors Model.

Subtest	Factor 1	Factor 2	Factor 3	Factor 4	Factor 5	Factor 6
Block Design	0.6423	0.1961	−0.4426			
Similarities	0.7732	−0.1772		0.1995	0.1832	
Digit Span	0.6856	0.1819	0.0668		−0.0810	−0.5084
MatrixReasoning	0.6974		−0.1989		−0.1067	
Vocabulary	0.8093	−0.1847	0.1148	0.1487	0.3281	
Arithmetic	0.7713			−0.2652		−0.1826
Symbol Search	0.5431	0.5508	0.1647			0.1394
Visual Puzzles	0.6401	0.1488	−0.4878			
Information	0.7189	−0.1617			0.3690	
Coding	0.6230	0.4108	0.2878		−0.2465	0.2077
L-N Sequencing	0.6504	0.1316				−0.4600
Figure Weights	0.7172		−0.2646	−0.1107		−0.1018
Comprehension	0.7808	−0.2289		0.2385	0.1446	
Cancellation	0.3584	0.4748				
PictureCompletion	0.4598	0.3426	−0.2770	0.1554	0.1390	

## Discussion

Models that describe subtests as multidimensional functions of uncorrelated factors provided a better fit to the WAIS-IV correlations than models that describe subtests as one-dimensional functions of correlated factors. This was true both for the WAIS-IV sample from which the free parameters were estimated as well as for the two samples from alternative populations to which these estimates were generalized. While accounting for the WAIS-IV correlations better, these multidimensional subtest models also had more free parameters.

Models with more free parameters may produce spurious results due to overfitting the data [25]. Fit indices that adjust for the number of estimated parameters have been proposed as a way to deal with this problem. However the continuing proliferation of these indices attests to the fact that there is no clear way to balance reduction in prediction error with number of estimated parameters. Since overfitting occurs when parameters account for chance variation in the data the most straightforward evaluation is through cross-validation [24], [26]. For all models in the present study only the residual variances were estimated for cross-validation, so that factor loadings could not be adjusted to chance variation in the test data. In the present study models were compared in terms of generalization of the parameters estimated from the 20–54 old samples to the 16–19 and 55–69 year old samples. These represent what Mosier [24] referred to as validity generalization since the new samples represent different populations rather than simply an additional sample from the same population. The generalization of the multidimensional models to new populations provides strong evidence that the superior fit of these models is not due to overfitting.

Overfitting is said to occur when parameters account for chance variation in the data [27]. There are several sources of chance variation in modeling the structure of cognitive abilities. These include error in sampling a given individual’s performance, sampling error within the population from which the parameters are estimated, error in sampling of alternative populations, and error associated with sampling of tests. Fit indices and crossvalidation deal with the error in parameter estimation associated with samples of finite size. Examples include the variability due to less than perfect test reliability and chance differences between the statistics of the sample and the statistics of the population from which it was drawn. If a model is to be used across a broad range of individuals, then the differences between the statistics of the population with which the parameters were estimated and these other populations represents a potential source of overfitting. That is, the model parameters may be tuned to idiosyncratic characteristics of one specific population. With tests such as the WAIS-IV the second order (i.e., subtest correlations) statistics are being modeled. The intended use of instruments such as the WAIS-IV includes a broad range of individuals in terms of age and abilities [11]. Consequently it is desirable to show that models generalize across these populations. The present study ruled out overfitting due to these first three factors by including evaluations of samples both younger and older than that used for parameter estimation. Consequently the superior fit of multidimensional models has broad generality across the populations used for standardization.

Model parameters might also be tuned to specific characteristics of the particular sample of tests that are used to estimate parameters. While the goal of many studies is to determine the structure of human abilities [12] the models obtained may be limited by the particular sample of tests modeled. In the present study, the model with six factors loading on all subtests provided the best fit on all of the indices in each sample. The 15 subtests available from the WAIS-IV do not provide sufficient degrees of freedom to evaluate a seven factor model with loadings on all subtests. However there is no reason to rule out the possibility that more than six factors might be useful in modeling human abilities. Thus the present study is limited in terms of the complexity of the model of abilities than could be evaluated. The manner in which abilities are sampled by specific subtests could also be an issue. For example, the WAIS-IV does not include tests of non-verbal memory span (e.g., [28]). There are no doubt many other types of tests that might be included. At present it is not clear how omission of such tests might bias estimates of the structure of human abilities (or alternatively how the generality of models might be restricted). Of all the sampling issues the impact of sampling tests is the most difficult to evaluate.

Some researchers discount the fact that models “fail” the X^2^ null hypothesis test. For example, in discussing their results, Benson and associates [12] state “*both models failed the* X^2^ *null hypothesis test of perfect fit. This does not mean that all aspects of either model are false. Rather, this finding merely refutes the claim that all aspects of either model are consistent with the data*.” While there are various rationales that might be offered for this attitude, it is important to appreciate the fact that models generally provide an incomplete account of the data. The fact that the value of X^2^ for the modified six orthogonal factor model was not significant does not indicate that it is the “true” model as it is probable that there are other models that would also not differ significantly from chance. The assumption that there is in fact a “true” model may be a fiction [25]. Rather the concern should be on accuracy and the ability of our models to generalize to a wide variety of different circumstances [29].

The advantage of a model with subtest performance determined by multiple factors is one of the key findings of the present study. The model summarized in Table 4 produced by modifying a “nested” version of the Benson and associates [12] model fit the training data and generalized to the test samples better than the three comparison models from the existing literature. Some of the resulting orthogonal factors could be interpreted roughly in the same manner as the original Benson and associates [12] factors with the exception that they loaded on multiple subtests. The pattern of these multiple loadings in many cases makes intuitive sense. For example, the Cancellation subtest requires that examinees search an array for one of two targets defined by a conjunction of visual features. The loadings in Table 4 for this subtest are on factors associated with Speed of Processing, Working Memory and Visual Processing. In addition to the Speed of Processing factor identified in the Benson and associates [12] model, Working Memory is involved to the extent that examinees must store both the task instructions and the targets in working memory. Indeed, there is experimental evidence for the role of working memory in visual search (e.g., [30]). In addition, the Cancellation subtest requires that multidimensional visual features must be identified, suggesting a role for Visual Processing. Limitations in any of these three factors would be expected to limit performance. As this example illustrates, the conceptualization of performance by models with simple structure may be too simple.

The model summarized in Table 5 produced by optimizing the six orthogonal factor model fit the data used to estimate parameter values better than all models except for the 6 orthogonal factor model from which it was derived and it generalized best to the older sample. There is some correspondence between the loadings for this model and the factors identified in the comparison models. However all of these factors except the general factor have loadings with mixed signs. There could be several explanations for this. This pattern could be a result of factor indeterminacy and might be eliminated by some rotation of the factors loadings. Alternatively it could reflect a real opposing relationship between different neural information processing modules as proposed, for example, by Grossberg [31]. Factor indeterminacy may be the most likely explanation (i.e., there are many models with equivalent fit). Modeling of simulated data using methods similar to that reported by McFarland [32] using six uncorrelated factors with positive weights on all of 15 tests produced a pattern of results similar to that in Table 5 (unpublished findings of the author). Although all six simulated factors were of equal magnitude, more variance was accounted for by a single general factor and the other five factors had weights with both positive and negative values. This pattern may be a characteristic of current factor analysis algorithms rather than of the processes generating the data.

While the models presented in Figures 4 and 5 are simpler in the sense that they have uncorrelated factors they are conceptually complex. In particular, it is unlikely that practicing clinicians would find several of the factors from Table 5 to be easily related to practice. However these results show that further improvements in modeling abilities are possible. Models such as those of Benson and associates [12] and Ward and associates [13], while conceptually simple, are not optimal. Even their conceptual simplicity may be misleading since, as discussed earlier, oblique and hierarchical factors are complex. The “nested” version of the Benson and associates [12] model overcomes this problem. However it may be too simple in that the models presented in Figures 4 and 5 provide better fits to the data. Conceptually it is not difficult to appreciate that performance on a given subtest might have multiple determinants. For example, Milberg and associates [3] have described multiple processes that might limit performance on single subtests of the WAIS-R. Application of a multiple process approach could lead to better models of human abilities.

The present study compared broad classes of models that can be characterized as one-dimensional with those that can be classified as multidimensional. The comparison models with simple structure are one-dimensional since, for the most part, each subtest loads on a single factor through which the effect of a general factor is mediated. Models that describe subtests as multidimensional functions of uncorrelated factors provided a better fit to the WAIS-IV correlations than models that describe subtests as one-dimensional functions of correlated factors. However it could be argued that one-dimensional models are more desirable because they are simpler. Along these lines, it is much easier to conceptualize each test as reflecting only a single underlying ability. However, as noted by Quine [33] simplicity is not easy to define and what is simple from one perspective may be complex from another perspective. In the present case, this simplicity in terms of subtest interpretation comes at the cost of increased complexity in the conceptualization of the latent traits. As discussed by Smith and associates [15], oblique and hierarchical models produce complex latent variables that do not represent homogeneous constructs. These models that are said to have a “simple structure” with a single factor loading on subtests use latent factors that are complex in the sense that they share covariance with the other factors in the model. Thus simplicity at the subtest level comes at the expense of complexity at the latent trait level.

Modifying the Benson and associates [12] model so that the general factor is orthogonal to produce what Gignac [14] called a nested model resulted in improved fit both in the sample used for estimating weights and in the samples used to evaluate generalization. In addition to improved fit, this model, as well as those summarized in Tables 4 and 5, has uncorrelated factors. As noted by Smith and associates [15] oblique and hierarchical models produce complex latent variables that do not represent homogeneous constructs. In addition, as pointed out by Gignac [14], hierarchical models imply that the effects of higher level factor are not direct, but rather are mediated by the group level factors. There are several implications of using oblique and hierarchical models. First of all, as discussed by Smith and associates [15], the use of homogeneous constructs can advance validation and diagnosis. In addition, studies such as those evaluating learning disabilities often control for intelligence [34]. This practice is questionable to the extent that the measure used to “control” for general ability reflects a mixture of heterogeneous tendencies. Finally there is a growing literature on biological correlates of human abilities [35]. Results of studies investigating the biological correlates of human abilities necessary depend on the way in which abilities are modeled.

The results of the present study also suggest that a useful model of cognitive test performance cannot be identified by the use of factor analysis in isolation. Constraining latent variable models with knowledge of neurophysiology and neuropsychology is probably a better strategy than constraining models by use of simple structure. In this regard, it is notable that Hampshire and associates [36] recently reported that several different brain networks contribute to distinct components of intelligence.

These results support the concept that subtest performance is determined by multiple orthogonal factors. In addition to the implications for modeling test performance, these results also support the concept that test behavior has multiple determinants [1], [21]. This contrasts to the view that each test has a single interpretation. This one-dimensional interpretation of test performance is not driven by experimental work but rather by the interpretation of the results of the application of simple structure to factor analysis [37]. As noted, experimental studies in cognitive science [38] and neuropsychology [39] suggest more complex determinants of constructs, such as short term memory, that are used in these models. The assertion that a single g factor accounts for most of the variance in cognitive test performance [4] may be based on the application of an overly simplistic model.

Future work could compare the extent to which simple structure and multidimensional models estimated from one sample of tests generalize to a different but related sample of tests. For example, Tulsky and Price [40] modeled the combined WAIS-III and WMS-III scales. Perhaps a better study of generalization would fix the weights computed from one of these batteries (e.g., the WAIS) when predicting the structure of the other. Other test batteries, such as the Woodcock-Johnson cognitive and achievement batteries could be modeled in a similar fashion. Such studies could address the question of generalization across scales.

In summary, models that describe subtests as multidimensional functions of uncorrelated factors provided a better fit to the WAIS-IV correlations than models that describe subtests as uni-dimensional functions of correlated factors. Although these models have more free parameters they generalize better to samples from different populations when parameter values are fixed. There may be a trade-off between simplicity of subtest modeling and complexity of latent factors. Consequently models employing multidimensional conceptualization of subtest performance should be explored further. Simple structure may be too simple.
